# Increased STAT3 Phosphorylation in CD4^+^ T-Cells of Treated Patients with Chronic Lymphocytic Leukemia and Changes in Circulating Regulatory T-Cell Subsets Relative to Tumor Mass Distribution Value and Disease Duration

**DOI:** 10.3390/biomedicines13051204

**Published:** 2025-05-15

**Authors:** Mojca Dreisinger, Zlatko Roškar, Aleš Goropevšek, Andreja Zakelšek, Sara Čurič, Nada Živko, Sebastjan Bevc, Evgenija Homšak

**Affiliations:** 1 Department of Haematology, University Medical Centre Maribor, 2000 Maribor, Slovenia; mojca.dreisinger@ukc-mb.si (M.D.); zlatko.roskar@ukc-mb.si (Z.R.); 2 Department of Laboratory Diagnostics, University Medical Centre Maribor, 2000 Maribor, Slovenia; ales.goropevsek@ukc-mb.si (A.G.); andreja.zakelsek@ukc-mb.si (A.Z.); sara.curic@ukc-mb.si (S.Č.); nada.zivko@ukc-mb.si (N.Ž.) evgenija.homsak@ukc-mb.si (E.H.); 3 Faculty of Medicine, University of Maribor, 2000 Maribor, Slovenia; 4 Department of Nephrology, University Medical Centre Maribor, 2000 Maribor, Slovenia

**Keywords:** chronic lymphocytic leukemia (CLL), signal transduction, cytokines, T-cells

## Abstract

**Introduction**: In mouse models of chronic lymphocytic leukemia (CLL), an effective anti-leukemia immune response was obtained by depleting a specific regulatory T-cell (Treg) subset. While STAT5 signaling could alter the homeostasis of naïve (nTreg) and activated (aTreg) subsets, which are capable of suppressing also CLL patients’ responses to microbial antigens, perturbed STAT3 signaling could drive CXCR5 expression in circulating T-follicular regulatory cells (Tfr) and their entry into the lymph node/tumor microenvironment. **Materials and Methods**: By using phospho-specific flow cytometry, we monitored STAT signaling/phosphorylation (pSTAT), in vitro responses to Sars-Cov2-antigen-specific stimulation, and circulating Treg subsets in relation to Binet stage and total tumor mass/tumor distribution (TTM/TD) scoring in 62 patients with CLL. **Results**: The percentage of CXCR5^+^ Treg significantly increased in patients with Binet stage B disease, and Tfr-like subsets were associated with higher TTM and lower TD. The pSTAT3 levels in CD4^+^ T-cells were only significantly increased in patients undergoing therapy. Lower nTreg percentages correlated with increased disease duration, and an increased aTreg/nTreg ratio correlated with SARS-CoV-2-antigen-induced STAT5 signaling responses. **Conclusions**: The results show increased amounts of circulating CXCR5^+^ Tfr-like subsets in patients with extensive lymph node involvement and augmented STAT3 signaling in patients on therapy. While STAT5 responses may drive nTreg differentiation into aTreg, nTreg decline is associated with increased disease duration.

## 1. Introduction

Chronic lymphocytic leukemia (CLL) is a cancer of mature, monoclonal B-lymphocytes, recognizable by their distinct immunophenotype. These cells accumulate in the bone marrow, lymphoid tissues, and bloodstream. CLL is the most common type of leukemia in Western countries [1]. Diagnosis requires at least 5 × 10^9^/L B-lymphocytes in the peripheral blood, maintained over a period of at least three months [2]. In contrast, small lymphocytic lymphoma (SLL) features fewer than 5 × 10^9^/L CLL-like cells in the blood but shows the involvement of lymph nodes or other tissues, often alongside bone marrow infiltration [3,4].

The distribution pattern of tumor cells has been shown to have prognostic value in CLL; this metric is categorized into three subtypes—pure leukemia, predominant leukemia, and predominant lymphoma—based on a tumor distribution (TD) value, and it correlates with treatment response and survival outcomes [5].

CLL/SLL is classified as an indolent (slow-growing) type of B-cell non-Hodgkin lymphoma (B-NHL) because it can remain asymptomatic for long periods. However, it may progress and transform into a more aggressive form, typically diffuse large B-cell lymphoma, in a process known as Richter transformation (RT) [4,6]. In addition, the so-called “accelerated CLL” is a rare and underdiagnosed entity that probably stands in the “gray zone” between CLL and RT, as a histologically aggressive CLL; in this middle form, an increase in size and proliferative activity of the CLL cells is found, as well as the expansion/confluence of the proliferation centers (PC) in the lymph nodes [7]. These characteristic structures are also found in typical cases of CLL. They are formed in lymphoid organs by the so-called proliferative compartment, which represents a small fraction of the entire CLL clone, while the vast majority of resting B cells are found in peripheral blood [7,8,9]. As immunohistological studies have also demonstrated, CLL cells in lymphoid organs show enhanced survival and proliferate in morphologically privileged sites with a specialized architecture of surrounding immune cells, namely, PCs, also called “pseudofollicles” [7,10]. These sites/tissues allow for close interactions between malignant cells and non-malignant/host cells, including both immune cells and the stromal cells constituting the tumor microenvironment (TME) [11,12].

Another type of specialized cells of stromal origin are the follicular dendritic cells (FDCs), which are located in the lymphoid germinal center [13]. Access to the FDCs, which is controlled by the CXCL13–CXCR5 chemokine signaling axis, confers proliferative stimuli upon the leukemia B cells, as demonstrated in the immunoglobulin heavy chain (IgH) enhancer T-cell leukemia-1 oncogene (Eμ-Tcl1) transgenic mouse model of CLL [14].

Another important player within the CLL microenvironment is nurse-like cells (NLCs), derived from monocytes and resembling macrophages [15]. A recent study described the activation and expansion of CXCR5-expressing T-follicular helper (Tfh) cells in co-cultures of NLCs and CLL cells [16]. Immune cells, including T-cell subsets, are altered within the TMEs of other B-NHLs and are implicated in malignant cell survival and drug resistance [17]. While malignant B cells of follicular lymphoma have been shown to depend on direct contact with Tfh cells, a factor that is essential in the formation and maintenance of the germinal center reaction [18], the development and progression of CLL is not merely related to intrinsic properties, such as the somatic mutations of leukemia cells, but rather involves complex interactions within the TME [19,20]. CLL cells actively shape the TME by secreting chemokines (e.g., CCL3, CCL4, and CCL22) that attract various populations of T-cells [21]. Tumor cells also reconfigure the functions of T-cells towards a leukemia-supportive and immunosuppressive microenvironment [11,21,22].

Lymph nodes from CLL patients have been shown to contain elevated numbers of regulatory T-cells (Tregs) and immunosuppressive CD4^+^ T-cell subpopulations and have been characterized by the expression of the specific transcription factor FOXP3 [12,23]. Tregs were first characterized by their high expression of CD25 (IL-2Ralha) and their ability to suppress proliferation and cytokine production in co-cultured effector T-cells [24]. In similar suppression assays, the ability to suppress T-cell responses when exposed to stimuli in vitro was also shown in Tregs from patients with CLL [25].

Originally recognized in mice for their role in preventing autoimmunity [26], Tregs have since been linked to the development of immunosuppressive environments in various human cancers. The association between peripheral-blood Tregs and poor prognosis in different cancers was found in previous studies [27]; in one study, a positive correlation was found between the total quantity of Tregs and the disease stage in patients with CLL [28]. While the mechanism by which Tregs contribute to tumor progression in CLL patients has not yet been established, the association between the activated subset of Tregs (aTregs) and the advanced disease stage strongly suggests a tumor-supporting/protective role for this specific subset of Tregs in disease [29]. In contrast to the resting/naïve population of Tregs (nTregs), which mostly produce TGF-b1, activated Tregs are a major source of IL-10, another cytokine commonly associated with immune suppression [30].

Nonetheless, the exact role of different Treg subsets and their cytokine signaling in disease progression and therapy response remains unclear. Additionally, while cytokine responses in CLL patients have been extensively studied in clonal B-cells, much less is known about their impact on CD4^+^ T-cells [31,32]. Cytokines like IL-10 and IL-6 influence both Tregs and conventional FOXP3-negative CD4^+^ T-cells (Tcons) via pathways that activate STAT3 proteins (Signal Transducers and Activator of Transcription 3), which then migrate to the nucleus and regulate gene expression involved in cell proliferation [33]. Recent studies have uncovered the unique roles of STAT3 in the differentiation of a specific subset of CXCR5-expressing Treg, so-called follicular regulatory T-cells (Tfr), implicated in regulating Tfh cells and the antibody response in germinal centers [13,34]. However, little is known about how STAT signaling dysfunctions in CD4^+^ T-cells might affect Treg/Tcon balance and contribute to CLL progression.

Our study aimed to examine whether Treg subsets and STAT phosphorylation (pSTAT) are altered in CD4^+^ T-cells from the blood of CLL patients and whether these changes correlate with disease stage (Binet classification), disease duration, tumor mass size, and distribution. We used phospho-specific flow cytometry to assess STAT signaling responses during in vitro SARS-CoV-2 antigen-specific stimulation and to analyze basal STAT3 activation in CD4^+^ T-cells.

## 2. Materials and Methods

### 2.1. Study Population

Sixty-two untreated patients who met the diagnostic criteria for CLL [35] were enrolled in this prospective, single-center cohort study. The disease burden was assessed using Binet staging [35,36].

A total of 39 out of 62 patients with the start of therapy at enrollment (disease activity according to the international workshop on CLL-iwCLL criteria [35]) were included in follow-up. CD4^+^ T-cell pSTAT3 levels were also determined for some patients during therapy. During the follow-up, a grade ≥3 infection episode was defined as in the Common Terminology Criteria for Adverse Events (CTCAE) Version 5.0. Demographic, clinical, and laboratory data at the follow-up study entry are presented in Table 1. Therapy during follow-up is described in Table 2.

Blood samples for the determination of Treg subsets and basal pSTAT3 levels were also collected from healthy adult volunteers with no history of allergy, acute infections, autoimmune diseases, or use of medications known to affect the immune system. The mean age of these individuals was 60.3 years (range: 51.4–82.7 years).

In addition to two patients enrolled in the initial part of the study, STAT5 signaling responses to antigen-specific stimulation were analyzed in nine additional CLL patients, irrespective of their disease stage. All had received two doses of the BNT162b2 mRNA COVID-19 vaccine 26 to 28 months prior to sampling, and a subset had subsequently recovered from SARS-CoV-2 infection within a few months. Relevant clinical and demographic data for these patients, who were not included in the follow-up cohort, are presented in Supplementary Table S1.

To assess STAT5 signaling responses following antigen-specific stimulation, blood samples were also obtained from ten healthy laboratory personnel 26 to 32 months after their second dose of the BNT162b2 mRNA COVID-19 vaccine or following recovery from SARS-CoV-2 infection.

Characteristics of the recruited healthy controls are shown in Supplementary Table S2.

### 2.2. Stimulation, Fixation, and Permeabilization for STAT Signaling Analysis

For the analysis of basal STAT3 phosphorylation ex vivo, samples were prepared from EDTA-anticoagulated whole blood. Phosphorylation was halted by adding 2 mL of a 5X dilution of BD Phosflow Lyse/Fix Buffer (BD Biosciences, San Jose, CA, USA) to 100 µL of whole blood in round-bottom 5 mL polystyrene tubes, followed by incubation at 37 °C in a water bath for 10 min.

Subsequently, samples were centrifuged at 300× *g* for 7 min, and cells were permeabilized by incubating them in 1 mL of ice-cold BD Perm Buffer III (BD Biosciences, San Jose, CA, USA) for 30 min.

### 2.3. Intracellular Staining and Flow Cytometry Analysis

Following permeabilization, cells were centrifuged at 300× *g* for 5 min and washed twice with 2 mL of phosphate-buffered saline (PBS). Cells were then stained for 30 min at room temperature in 100 µL of staining buffer (PBS containing 2% fetal bovine serum) using the following antibodies: anti-CD45-PerCP (5 μL, clone 2D1), BV786 (1 μL, clone HI30) or APC-Cy7 (5 μL, clone 2D1), anti-CD3-FITC (10 μL, clone UCHT1), BV650 (3 μL, clone SK7), or PerCP (20 μL, clone SK7), along with antibodies targeting phosphorylated STAT3 at tyrosine 705: pSTAT3 (Y705)-Alexa488 (10 μL, clone 4) (all BD Biosciences, San Jose, CA, USA). For multiparametric immunophenotyping experiments, cells were simultaneously stained at room temperature with antibodies specific for T-cell-subsets: anti-CD4-PECy7 (5 μL, clone SK3) or BV750 (2 μL, clone SK3); anti-CD25-PE (10 μL, clone 2A3), BV421 (3 μL, clone 2A3), or APC (5 μL, clone 2A3); CD45RA PE-Cy7 (0.5 μL, HI100) or APC (5 μL, clone HI100); anti-Ki67 PE (10 μL, clone B56) or BV650 (3 μL, clone B56); and anti-FOXP3-Alexa 488 (10 μL, clone 259D/C7) or PE (10 μL, clone 259D/C7) (all BD Biosciences, San Jose, CA, USA). After a final wash with 2 mL of staining buffer, cells were analyzed on either an LSR II or a FACSymphony A3 flow cytometer (BD Biosciences, Franklin Lakes, NJ, USA) using FACSDiva software version 9.0 (BD Biosciences) and FlowJo software version 10.8 (TreeStar, Ashland, OR, USA, now part of BD Biosciences). Median fluorescence intensity (MFI) of the pSTAT3-specific signal was measured.

### 2.4. 13 Colour Surface Immunophenotyping

When Treg subpopulations were determined based on surface antigens in a 13-colour panel, the following antibodies were used simultaneously: anti-CXCR5 (CD185)-BB515 (3 µL, clone RF8B2), anti-CD161 PE (10 µL, clone DX12), anti-CCR7 (CD197) PE-CF594 (3 µL, clone 2-L1-A), anti-HLA-DR PerCP-Cy5. 5 (10 µL, clone L243), anti-CD45RA-PECy7 (0.5 µL, clone HI100), anti-CD127-Alexafluor647 (10 µL, clone HIL-7R-M21), anti-CD38-APC-H7 (3 µL, clone HB7), anti-CD25-BV421 (3 µL, clone 2A3), anti-CD15s-BV510 (3 µL, clone CSLEX1), anti-CD31-BV605 (3 µL, clone WM59), and anti-CD28-BV711 (3 µL, clone CD28).

Aliquots of whole blood (100 µL) from EDTA tubes were treated with 2 mL of 10x diluted FACSlyse erythrocyte lysis buffer (BD Biosciences) after 15 min of labeling with the described monoclonal antibodies in the dark. The samples were then centrifuged at 300× *g* for 5 min and washed with 2 mL of Stain Buffer BSA (BD Pharmingen/BD Biosciences, San Jose, CA, USA). Finally, the samples were centrifuged again at 300× *g* for 5 min, and 500 µL of the same Stain Buffer was added prior to analysis with a FACSymphony A3 Flow Cytometer.

To establish gating for CD38⁺ cells and to compare histograms of programmed cell death protein 1 (PD-1) expression, a fluorescence-minus-one (FMO) control was employed. In these controls, the same staining panel was used as in the complete sample, omitting only the anti-CD38 or anti-PD-1 antibody, respectively.

### 2.5. Unsupervised Analysis by Flow Cytometry

Cytometry data acquired from blood samples of 10 CLL patients and 10 healthy controls (HC) were first analyzed/gated in FlowJo software to remove debris and doublets via FSC/SSC discrimination. Then, gating on CD3^+^CD4^+^CD25^+^CD127^lo/−^ Treg cells was performed, and the same number of events in each sample was pooled as representative of the whole sample using the FlowJo Downsample algorithm. UMAP (Uniform Manifold Approximation and Projection) dimensionality reduction algorithm was used, which works similarly to tSNE (t-distributed stochastic neighbor embedding). However, UMAP has no computational restrictions on the embedding dimension, and it is more competent at preserving the global structure of the data. The number of metaclusters was defined by first running X-shift plugin/algorithm. The FlowSOM clustering algorithm was used for final hierarchical clustering to sort all events into a specified number of metaclusters.

### 2.6. Flow Cytometric Analysis of STAT Phosphorylation in Treg Subsets After Whole-Blood Stimulation with SARS-CoV-2-Specific Antigens

To evaluate STAT5 signaling responses to antigen-specific stimulation, whole blood collected in heparinized antigen tubes from the QuantiFERON SARS-CoV-2 kit (Qiagen, Hilden, Germany) was utilized. The SARS-CoV-2 Ag1 tube contains epitopes from the S1 subunit of the spike protein, targeting CD4⁺ T-cells, whereas the Ag2 tube includes epitopes from both the S1 and S2 subunits, targeting CD4⁺ and CD8⁺ T-cells.

Immediately after collection, samples were gently agitated and incubated at 37 °C for 16–24 h. Aliquots (120 μL total) were then collected from the Nil tube (negative control) and the two antigen tubes (60 μL each, pooled) prior to centrifugation and preparation for STAT signaling analysis. In selected experiments, a neutralizing anti-IL-2 antibody (2 μg/mL, clone MQ1-17H12, BD Biosciences) was added and incubated for 20 min at 37 °C prior to fixation.

Phosphorylation was halted by adding 2 mL of BD Phosflow Lyse/Fix Buffer (BD Biosciences, San Jose, CA, USA) to the samples and incubating for 10 min. Samples were then centrifuged at 300× *g* for 7 min and permeabilized via incubation with 1 mL of ice-cold BD Perm Buffer III (BD Biosciences, San Jose, CA, USA) for 30 min. Following permeabilization, cells were centrifuged at 300× *g* for 5 min and washed twice with 2 mL of phosphate-buffered saline (PBS).

Subsequently, cells were stained for 30 min at room temperature in 100 μL of staining buffer (PBS with 2% fetal bovine serum) with a combination of anti-human fluorescent monoclonal antibodies (all from BD Biosciences): FOXP3-FITC or PE, CD4-BV750, CD45RA-PECy7 (3 μL, clone HI100), CD25-BV421, and CD3-BV786 (2 μL, clone UCHT1). Additionally, antibodies targeting specific phosphorylated STAT tyrosines were used: pSTAT3 (Y705)-Alexa488 and pSTAT5 (Y694)-Alexa647 (10 μL, clone 47).

Flow cytometric analysis was conducted on a FACSymphony A3 (BD Biosciences), and data were processed using FlowJo software version 10.8 (TreeStar) and FACSDiva software version 9.0 (BD Biosciences).

### 2.7. Statistical Analysis

Statistical analyses were conducted using GraphPad Prism version 10 for Windows (GraphPad Software, San Diego, CA, USA). Between-group comparisons were performed using the Mann–Whitney test, while within-group comparisons were assessed using the Wilcoxon matched-pairs signed rank test. Spearman’s correlation coefficient was calculated to evaluate potential associations between variables. A *p*-value of less than 0.05 was considered statistically significant. The Kaplan–Meier method was employed to analyze the disease course and infectious complications during the follow-up period, with group differences assessed using the log-rank test. Comparisons of clinical outcomes and associated variables between subgroups were performed using the appropriate non-parametric tests, specifically the Mann–Whitney or Fisher’s exact test. Where applicable, the Bonferroni correction was applied to adjust for multiple comparisons.

## 3. Results

### 3.1. Increased Frequency of T Follicular Regulatory Subset in Patients Is Associated with Total Tumor Mass Score

**Figure 1:**
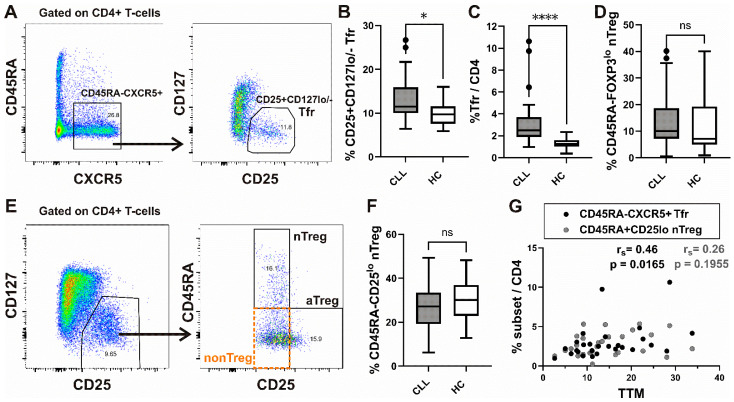
Increased frequency of T_fr_ subset in peripheral blood from CLL patients and correlation with total tumor mass (TTM) scoring. (**A**) Gating hierarchy for identification of CXCR5^+^CD45RA^−^ cells among CD4^+^ T-cells and CD25^+^ CD127^low/−^ T follicular regulatory cells (Tfr) among them; (**B**,**C**) Box-and-whisker plots show the percentage of CD25^+^ CD127^low/−^ Tfr cells among CD45RA^−^CXCR5^+^ CD4^+^ T-cells and Tfr among CD4^+^ T-cells from patients with CLL (*n* = 27) and HCs (*n* = 20). (**D**) The percentage of nTreg, defined as CD45RA^+^ FOXP3^lo^ cells among gated FOXP3^+^CD4^+^ T-cells, as shown on Supplementary Figure S1, from patients with CLL (*n* = 54) and healthy controls (*n* = 20). (**E**) CD25^+^ CD127^lo^/^−^ Treg cells among gated CD4^+^ T-cells were also subdivided into three fractions based on CD45RA and the level of CD25 expression: (I) CD45RA^+^ CD25^lo^ nT_reg_, (II) CD45RA^−^ CD25^hi^ aT_reg_, and (III) CD45RA^−^ CD25^lo^ non-T_reg_ subsets, as shown in representative dot plot. (**F**) The percentage of nTreg, defined as CD45RA^+^ CD25^lo^ cells among CD25^+^ CD127^lo^/^−^ Treg from patients with CLL (CLL, *n* = 28) and healthy controls (HC, *n* = 20). (**G**) Correlation between TTM score and percentage of Tfr among CD4^+^ T-cells (black symbols, *n* = 27) and CD45RA^+^ CD25^lo^ nT_reg_ among CD4^+^ T-cells (gray symbols, *n* = 27) in patients with CLL. *r_s_*, Spearman correlation coefficient; ns, not significant; **** *p* < 0.0001; * *p* < 0.05.

When we first analyzed CD45RA-CXCR5^+^ cells in peripheral blood, the frequency of CD25^+^CD127^low/−^ Tfr among them and CD4^+^ T-cells was significantly increased in our patients with CLL compared to HC (Figure 1A–C).

Next, Treg analysis was performed by using the strategy introduced by Miyara et al. [30], allowing for functional delineation of FOXP3/CD25 high-expressing CD45RA^−^FOXP3^hi^/CD25^hi^ activated Treg (aTreg) subset and the two FOXP3/CD25 low-expressing subsets: CD45RA^+^FOXP3^lo^/CD25^lo^ resting or naïve Treg (nTreg), and the CD45RA^−^FOXP3^lo^/CD25^lo^ (non-Treg) subset (Figure 1E and Supplementary Figure S1A). When we analyzed the expression of C-X-C chemokine receptor type 5 (CXCR5), which drives T follicular regulatory (Tfr)-like features in Treg cells [37], we found a significant increase in CXCR5^+^ cells among non-Treg and nTreg, as well as the aTreg subset, compared to HC. The highest percentage of CXCR5^+^ cells was found in the nTreg subset from both CLL patients and HC (Supplementary Figure S1B). However, the percentage of the latter subset defined as either CD45RA^+^CD25^lo^ or CD45RA^+^FOXP3^lo^ cells among CD4^+^ T-cells was not significantly different between CLL patients and HC (Figure 1D,F). When Treg cells were defined as CD25^+^CD127^lo/−^ (Figure 1E, left) cells, their percentage among CD4^+^ T-cells from our patients with CLL was significantly correlated with the percentage of both Treg subsets combined (aTregs plus nTregs) (Supplementary Figure S1C).

To evaluate the tumor burden across all major body compartments, we applied the total tumor mass (TTM) scoring system in our cohort of CLL patients [38]. The TTM score is calculated as the sum of (1) the square root of the peripheral blood lymphocyte count per nL, (2) the diameter of the largest palpable lymph node in centimeters, and (3) the extent of splenic enlargement measured below the left costal margin in centimeters.

In our study population, a significant correlation was observed between the TTM score and the proportion of Tfr cells among CD4⁺ T-cells, whereas no such correlation was detected with the proportion of nTreg cells (Figure 1G).

### 3.2. Unsupervised Cell Clustering Analyses Show Increased CXCR5 Expressing Populations in CLL Treg Cells and Alterations in Their Phenotypes

**Figure 2:**
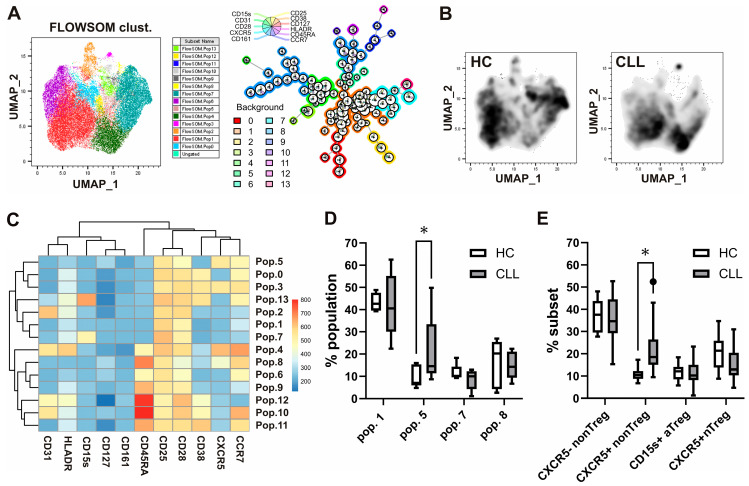
Alterations in Treg phenotypes and increased CXCR5-expressing populations in patients with CLL identified via unsupervised cell clustering analyses and confirmed by standard gating. (**A**) Cytometry data acquired from blood samples of 10 CLL patients and 10 HC were gated in Flowjo software, and equal numbers of CD25^+^ CD127^lo/−^ Treg cells from CLL patients and healthy controls were introduced in the analysis. A total of 14 phenotypically distinct populations within Treg cells identified using FlowSOM clustering algorithm are shown. (**B**) Differences in the distribution of clustered populations as evidenced by the UMAP analysis. (**C**) Populations were identified and clustered by FlowSOM according to their level of expression of cell surface markers CD25 (IL-2Rα) and CD127 (IL-7Rα), as well as CCR7, CXCR5, CD28, CD38, CD161, CD31, CD45RA, HLA-DR, and CD15s. (**D**) The percentages of the four populations, identified using unsupervised clustering, were represented by at least 1% of Treg cells from patients with CLL and HC. (**E**) Percentages of corresponding subsets among Treg cells via standard gating from patients with CLL and HC. * *p* < 0.05.

Standard gating is limited to analyzing only two markers at a time in a flow plot. Additionally, the markers’ expression levels are often not considered, opting for the simpler distinction between “positive” and “negative” populations. As an alternative, dimensionality reduction analyses were carried out using the UMAP and FlowSOM cell clustering algorithms from FlowJo V10.8. UMAP allows for clustering cells depending on their expression levels of multiple markers in a two-dimensional (2D) space. FlowSOM identifies phenotypically distinct populations that can be visualized in the UMAP 2D output. Combining these tools, we were able to analyze the phenotype of populations over- or under-represented in CLL, compared to controls, within Treg cells, defined as CD25^+^ and CD127 low-expressing/- CD4^+^ T-cells, as shown in Figure 1E. Equal numbers of CD25^+^ CD127^lo/−^ CD4^+^ T-cells from CLL patients and healthy controls were introduced in the analysis and clustered according to their level of expression of cell surface markers CD25 (IL-2Rα) and CD127 (IL-7Rα), as well as CCR7, CXCR5, CD28, CD38, CD161, CD31, CD45RA, HLA-DR, and CD15s. FlowSOM identified 14 phenotypically distinct populations within Treg cells (Figure 2A). The UMAP analysis evidenced differences in the distribution of clustered populations, with some being over or under-represented in CLL (Figure 2B), while being clustered according to their level of expression of the analyzed cell surface markers (Figure 2C). Among the four populations, which were represented with at least 1% cells among Treg (Figure 2D), the most prevalent in both CLL patients and HC was the CD45RA^−^CD25^low+^ non-Treg-like population, which lacked expression of other markers, including CXCR5. The increased population in CLL compared to HC was the CXCR5^+^ Tfr-like CD45RA^−^ memory phenotype, which could also be »hidden« in the non-Treg subset. On the other hand, the population with the CXCR5^+^ Tfr naïve or resting-like CD45RA^+^ phenotype and the population that had characteristics of CD15s^+^ effector-like aTreg cells were not significantly different compared to healthy controls. This is related to the result observed by standard gating (Figure 2E), where we observed a significant increase only in the CXCR5^+^ subset of non-Treg, but not CXCR5^−^ non-Treg, CD15s^+^ aTreg, or CXCR5^+^ nTreg in CLL patients compared to HC. These results suggested that memory, but not the naïve subset of T follicular regulatory cells like Tregs, may be expanded explicitly in CLL.

### 3.3. The Increase in CXCR5^+^ Subset of Treg Cells in Patients with CLL Correlates with Tumor Mass Distribution Value

Next, Treg subsets from patients with CLL were analyzed in relation to the tumor distribution (TD) assessment, which is based on the TTM scoring system [38], where the TD value represents the percentage of total tumor mass infiltrating peripheral blood and bone marrow (TD = TM(1)/TTM).

CLL can be categorized into three subgroups: pure leukemia when TD100%, predominant leukemia if TD = 50–99%, and predominant lymphoma when TD < 50% [5].

**Figure 3:**
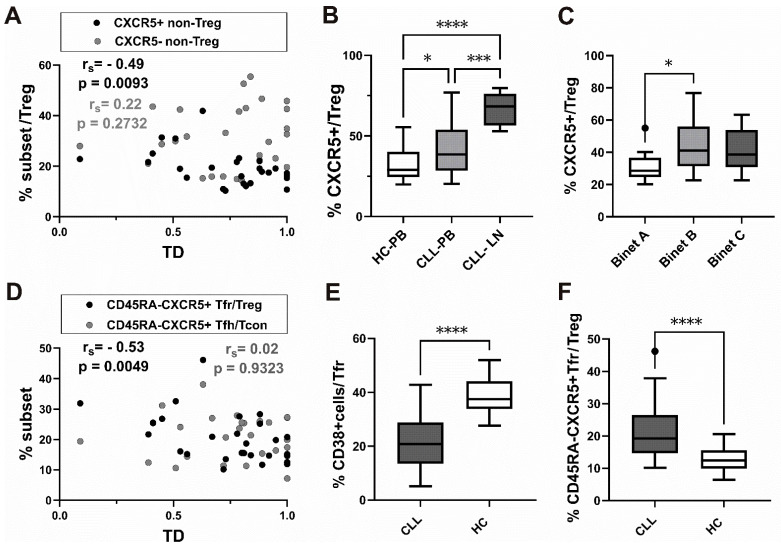
Association of CXCR5^+^ subsets of Treg cells with tumor mass distribution (TD), Binet staging, and decrease in CD38^+^ subset among CD45RA-CXCR5^+^ Tfr cells from patients with CLL. (**A**) Correlation between TD values and percentage of CXCR5^+^ non-Treg among CD4^+^ T-cells (black symbols, *n* = 27) and CXCR5^−^ non-Treg among CD4^+^ T-cells (gray symbols, *n* = 27). (**B**) Box-and-whisker plot shows the percentage of CXCR5^+^ cells among gated CD25^+^ CD127^lo/−^ Treg-cells in peripheral blood from patients with CLL (*n* = 34) and healthy controls (*n* = 20) (**C**) The percentage of CXCR5^+^ cells among gated CD25^+^ CD127^lo/−^ Treg-cells in peripheral blood from CLL patients with Binet stage A (*n* = 10), Binet stage B (*n* = 10), and Binet stage C (*n* = 14) disease. (**D**) Correlation between TD values and percentage of CXCR5^+^CD45RA^−^ Tfr among CD25^+^CD127^lo/−^ Treg (black symbols, *n* = 27) and percentage of CXCR5^+^CD45RA^−^ Tfh among CD25^−^CD127^+^ Tcon (gray symbols, *n* = 27). (**E**) The percentage of CD38^+^ cells among gated CD45RA-CXCR5^+^CD25^+^ CD127^lo/−^ Tfr-cells in peripheral blood from patients with CLL (*n* = 25) and healthy controls (*n* = 20). (**F**) The percentage of CD45RA^−^CXCR5^+^ Tfr cells among gated CD25^+^ CD127^lo/−^ Treg-cells in peripheral blood from patients with CLL (*n* = 28) and healthy controls (*n* = 20). *r_s_*, Spearman correlation coefficient; ns, not significant; * *p* < 0.05, *** *p* < 0.001, **** *p* < 0.0001.

We found a significant negative correlation between TD values and an increased percentage of CXCR5^+^ non-Treg, but not CXCR5^−^ non-Treg, among CD4^+^ T-cells (Figure 3A).

In addition, CLL peripheral blood samples showed a significantly higher percentage of CXCR5^+^ cells among Treg than blood samples from healthy controls (Figure 3B).

As we observed that the percentage of CXCR5^+^ cells in the Treg subset was increased in peripheral blood from patients with untreated CLL, we then went on to see how CXCR5^+^ Treg correlated with the stage of the disease. The Binet classification assigns patients with CLL to one of three groups, A–C, based on the extent of disease. When we analyzed the CXCR5^+^ subset in patients at different stages of disease, those with untreated stage B and C disease had a higher percentage of CXCR5^+^ cells among CD25^+^CD127^lo+/−^ Treg cells compared to stage A disease. The increase was significant in stage B, characterized by three or more areas of enlarged lymphoid tissue (Figure 3C).

Finally, a significant negative correlation between TD values and the Tfr defined as CXCR5^+^CD45RA^−^ cells among Treg, but not CD45RA^−^CXCR5^+^ Tfh among CD25^−^CD127^+^ Tcon, was found (Figure 3D).

Therefore, increased frequencies of both the CXCR5^+^ non-Treg subset and CXCR5^+^CD45RA^−^ Tfr among CD25^+^CD127^lo/−^ Treg cells were found in patients with CLL associated with lower TD values, representing predominant lymph node involvement.

Negative correlation between this value and CXCR5^+^ expressing Tfr-like subsets suggests that the latter subsets are expanded in peripheral blood from CLL patients with more lymphoma-like disease.

### 3.4. CD38^+^ Subset Is Decreased in CXCR5^+^CD45RA^−^ Tfr from CLL Patients

Both Tfh and Tfr, crucial in generating and regulating the antibody response, exert their function primarily in lymph nodes/secondary lymphoid organs. Nevertheless, recent studies in animal models of SARS-CoV-2 and after vaccination against other viral pathogens in humans have shown that the detection of activated CD38^+^ Tfh-like cells in peripheral blood at an appropriate early time window can predict a subsequent effective antibody response [39,40].

When we analyzed the percentage of CD38^+^ cells among CD45RA^−^CXCR5^+^ Tfr in patients with CLL, it significantly decreased compared to HC (Figure 3E).

Of note, while PD-1 expression was significantly higher in CD45RA^−^CXCR5^+^ Tfh from patients with CLL compared to HC, CD45RA^−^CXCR5^+^ Tfr, which were significantly increased among Treg in patients (Figure 3F), displayed significantly lower PD-1 expression compared to HC (Supplementary Figure S2A–C).

### 3.5. Higher Basal STAT3 Phosphorylation Levels in CD4 T-Cells from Patients with CLL Treated with Ibrutinib or Chemo-Immunotherapy

**Figure 4:**
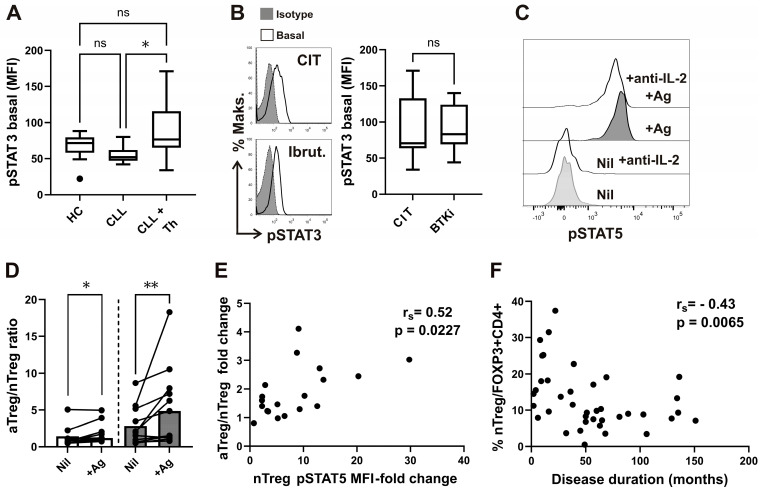
Elevated basal pSTAT3 levels in CD4^+^ T-cells of CLL patients undergoing therapy and correlation of disease duration with percentage of nTreg subset. (**A**) Box-and-whisker plot shows basal pSTAT3 levels (MFI) in CD4^+^ T-cells from samples of untreated patients with CLL (CLL, *n* = 16), patients with CLL on CIT or therapy with BTKi (CLL+Th, *n* = 22), and HCs (*n* = 15). (**B**) Representative histograms of pSTAT3 in gated CD4^+^ T-cells from sample of patients with CLL on CIT and patients with CLL on therapy with BTKi-Ibrutinib are shown left: basal, untreated cells are compared with cells stained with isotype control. pSTAT3 levels (MFI) in CD4^+^ T-cells from samples of patients with CLL on CIT (CIT, *n* = 12) and therapy with BTKi (BTKi, *n* = 10). (**C**) pSTAT5 levels in whole blood samples were collected and incubated/stimulated in tubes from Quantiferon SARS-CoV-2 kit for 16–24 h, as described in the Materials and Methods. Representative histograms of pSTAT5 in gated CD25^+^FOXP3^+^CD4^+^ T-cells from the same healthy donor whole-blood aliquots withdrawn from negative control (Nil) and from the two Ag tubes mixed together (+Ag), incubated with anti-IL2 neutralizing antibodies or left untreated, are shown. (**D**) aTreg/nTreg ratio in whole-blood aliquots withdrawn from negative control (Nil) and from the Ag tubes (+Ag). Symbols represent samples from healthy donors (white bars) and patients with CLL (gray bars). (**E**) Correlation between pSTAT5 MFI fold change (pSTAT5 MFI in stimulated tube divided by pSTAT5 MFI in control tube) and the increase in fold change in aTreg/nTreg ratio. (**F**) Correlation between percentage of nTreg among CD4^+^FOXP3^+^ T-cells from patients and disease duration (*n* = 39). *r_s_*, Spearman correlation coefficient; ** *p* = 0.001–0.01; * *p* < 0.05.

As STAT3 signaling is implicated in CXCR5 expression [41], STAT3 phosphorylation (pSTAT3) levels were first compared between CD4^+^ T-cells from untreated CLL patients, CLL patients receiving therapy at the time of analysis, and healthy donors. CD4^+^ T-cell pSTAT3 median fluorescence intensity (MFI) was not significantly different from HC. However, pSTAT3 MFI increased dramatically in treated CLL patients compared to patients not receiving therapy (Figure 4A). When a group of patients receiving BTKi during follow-up was compared to patients receiving chemo-immunotherapy (CIT), no significant differences in pSTAT3 levels were found (Figure 4B).

### 3.6. STAT Signaling and Treg Changes During SARS-CoV-2 Antigen-Specific Stimulation

We also assessed STAT signaling responses following antigen-specific stimulation using heparinized antigen tubes from the QuantiFERON SARS-CoV-2 kit, which enable simultaneous whole-blood collection and stimulation of lymphocytes with a combination of two SARS-CoV-2-specific peptide antigens (Ag1 and Ag2). The SARS-CoV-2 Ag1 tube contains epitopes derived from the S1 subunit of the spike protein recognized by CD4⁺ T-cells, whereas the Ag2 tube contains epitopes from both the S1 and S2 subunits recognized by CD4⁺ and CD8⁺ T-cells.

Whole-blood samples from healthy donors who had received the BNT162b2 mRNA COVID-19 vaccine and/or recovered from SARS-CoV-2 infection were analyzed. Additionally, samples from 11 CLL patients who had received two doses of the BNT162b2 mRNA COVID-19 vaccine were examined; some of these patients had also recovered from recent SARS-CoV-2 infection and tested positive for anti-Spike antibodies.

We evaluated STAT signaling and Treg subsets in whole-blood aliquots obtained from the Nil (negative control) tube and the combined Ag1 and Ag2 tubes of the QuantiFERON SARS-CoV-2 kit.

While pSTAT5 levels in CD25⁺FOXP3⁺ Tregs were elevated in antigen-stimulated tubes compared to control tubes, pSTAT3 levels remained unchanged between the conditions (Figure 4C and Supplementary Figure S3).

Although the QuantiFERON SARS-CoV-2 assay is primarily a whole-blood interferon-gamma release assay (IGRA), IL-2 is also produced during antigen-specific T-cell activation.

Indeed, the observed increase in pSTAT5 levels in antigen-stimulated tubes was IL-2-dependent, as demonstrated by their reduction upon treatment with neutralizing anti-IL-2 antibodies (Figure 4C).

Furthermore, antigen-specific stimulation altered the homeostasis of phenotypically suppressive Treg subsets, as evidenced by a significant increase in the aTreg/nTreg ratio in both CLL patients and healthy controls (HC) in antigen-stimulated (+Ag) tubes compared to Nil controls. Although the aTreg/nTreg ratio was already elevated in Nil tubes, the increase following antigen stimulation was even more pronounced in samples from CLL patients (Figure 4D).

Moreover, STAT5 signaling responses within the CD45RA⁺FOXP3^lo nTreg subset, assessed as the fold change in pSTAT5 MFI (stimulated/control), significantly correlated with the fold change in the aTreg/nTreg ratio (Figure 4E). Thus, the antigen-specific increase in the aTreg/nTreg ratio was associated with IL-2-dependent STAT5 signaling, but not with STAT3 signaling.

These findings suggest that IL-2-dependent STAT5 signaling responses elicited by antigen-specific stimulation may facilitate the differentiation of nTreg into aTreg.

### 3.7. Lower nTreg Frequencies Correlate with Longer Disease Duration

In our previous study, we observed that enhanced STAT5 signaling—which may promote the conversion of naïve regulatory T-cells (nTregs) to activated regulatory T-cells (aTregs)—was present in treated patients. Additionally, a higher proportion of aTregs was linked to an increased frequency of serious infections during follow-up. Based on these findings, we aimed to determine whether CLL patients with lower percentages of nTregs among FOXP3^+^CD4^+^ T-cells at the beginning of follow-up would experience a more severe disease progression, specifically in the form of infections requiring hospitalization. We employed the previously described methodology [29].

The study cohort included 39 CLL patients, most of whom were in stage C before starting treatment. Patients were categorized into subgroups based on their baseline nTreg percentages (see Table 1) and followed for up to 500 days. We found that by day 365, 42% of patients in group 2—those with lower nTreg frequencies (<9.6%)—developed grade ≥3 infections, compared to 25% in group 1, who had higher nTreg frequencies (≥9.6%). However, Kaplan–Meier survival analysis with the Log-rank test did not show a statistically significant difference between the two groups (Supplementary Figure S4A). Furthermore, the average rate of grade ≥3 infections per year of follow-up was not significantly different between the groups (0.50 per year in group 2 vs. 0.33 per year in group 1, *p* = 0.6249).

During the follow-up, five patients were hospitalized due to severe COVID-19: two from group 1 (higher nTreg levels) and three from group 2 (lower nTreg levels), as shown in Supplementary Figure S4B.

No significant associations were observed between infection rates and type of therapy (Table 2) or other clinical or laboratory parameters, except for disease duration. Group 2 patients, with lower nTreg frequencies, had a significantly longer disease duration compared to those in group 1, although this significance did not hold after Bonferroni correction (Table 1). Consistently, we found a significant negative correlation between the percentage of nTregs among CD4^+^FOXP3^+^ T-cells and disease duration (Figure 4F). This correlation was specific to the nTreg subset, as the percentage of aTregs showed no significant correlation with disease duration (rs = 0.03, *p* = 0.8498).

## 4. Discussion

Recent results in the mouse model of CLL indicate that an effective anti-leukemia immune response can be obtained by depleting a specific Treg subset in the CLL microenvironment, thereby creating an opportunity to expand a population of cytotoxic CD8^+^ T-cells. Such a specific Treg population was formed during the progression of TCL1 leukemia [37].

Also, patients with CLL Treg subsets identified by the 15-color immunophenotyping were associated with prognostic markers/predictors of disease progression [42].

The Tfr subset significantly increased in CD4^+^ T-cells in our patients compared to HC. The same subset, but not nTreg, was significantly associated with the TTM score, indicating the tumor mass within all major body compartments.

Compared to HC, a significantly higher percentage of not only the non-Treg fraction but also nTreg and aTreg subsets from our patients expressed the key chemokine receptor CXCR5, which allows access to B-cell follicles in lymph nodes [13].

Therefore, increased expression of CXCR5 on both suppressive subsets in patients with CLL could support entry into TME of functional Treg subsets, promoting tumor immune evasion by suppressing antitumor T-cell responses not only through a contact-dependent (cytotoxic T-lymphocyte-associated protein 4/CTLA-4 expression in aTreg cells) but also cytokine-dependent mechanism [43,44].

In our patients, nTreg and aTreg subsets significantly correlated with the percentage of Treg defined as CD25^+^CD127^low+/−^ cells. The latter suggests that nTreg and aTreg comprise phenotypically suppressive FOXP3^+^ cells in our patients with CLL, as T_reg_-suppressive function is linked to both low expression of CD127 and high surface expression levels of CD25 [45,46]. In line with this, in our previous study, significantly lower expression of CD25 was found in the non-Treg fraction from patients with CLL compared to both nTreg and aTreg subsets [29].

When investigating the potential role of human FOXP3^+^ Treg cells in immune suppression, it is critical to analyze phenotypically suppressive subpopulations, such as nTreg and aTreg, and not only total FOXP3^+^CD4^+^ T-cells, as they also contain the non-Treg fraction, which has reduced in vitro suppressive activity. It also contains cells that produce inflammatory cytokines and is phenotypically characterized by both low FOXP3 and low CD25 expression [30]. However, recent results showed that while CD127^+^CD25^lo^ cells in the non-Treg fraction have a high level of inflammatory cytokine production, CD127^−^CD25^lo^ cells may be best understood as a mixture of non-Treg and Treg cells [44). In addition, the CD45RA^−^CXCR5^+^CD25^lo^ circulating T-follicular regulatory (Tfr) subset is also present in the non-Treg fraction. It has stable Foxp3 expression and suppressive function, confirming that the population, which makes up around 30% of the cells in the non-Treg fraction in the blood of healthy donors, is composed of Treg cells [44].

Among 14 phenotypically distinct populations of Treg cells, identified by 13-color immunophenotyping and unsupervised FlowSOM clustering, population 5, which was more abundant in our CLL patients, was CXCR5-positive and showed a CD45RA negative-memory phenotype similar to CXCR5^+^ Tfr cells. It could also correspond to non-Treg cells by slightly lower CD25 expression levels. The results of such algorithmic cell analysis were then confirmed by standard cell gating, as we found a significantly higher proportion of the CXCR5^+^ subpopulation corresponding to Tfr cells with a CD45RA^−^CD25^low+^ phenotype of non-Treg cells in CLL patients compared to controls. The so-called non-Treg cell population is thus probably best understood in the context of CLL as a heterogeneous group of cells in the peripheral circulation containing not only cells without suppressor function and with the ability to produce proinflammatory cytokines (IL-17 and IFN-g), but also circulating CXCR5^+^ T regulatory follicular cells (Tfr), perhaps reflecting the continuous production of (auto)antibodies [44].

In addition, the higher proportions of CXCR5^+^ non-Treg cell populations among CD4^+^ T-cells from patients with CLL were significantly associated with a lower tumor distribution (TD) value, predominantly representing lymph node involvement. Tfr, a subset of Tregs expressing transmembrane chemokine receptor CXCR5, plays a major role in suppressing the immune response in secondary lymphoid follicles [47]. CXCR5^+^ Tfh and Tfr cells may be divided into CCR7^+^ perifollicular cells and CCR7^−^ follicular cells [48]. According to recent studies, Tfr shows the most effective suppressive activity in humans at the interface between the B cell follicle and the T lymphocyte region and within the follicle, but not in the GC itself [49]. However, more studies are required to investigate the Tfr function within the CLL proliferation centers of the lymph nodes. Human Tfr does not originate entirely from the thymus but partly arises by differentiation from Tfh. Tfr originating from Tfh precursors was recently shown to express CD38 [50]. Of note, population 5, identified by FlowSOM clustering, which was more abundant in our CLL patients, expressed CCR7 and was also characterized by the lack of CD38 expression, suggesting that it may not be derived from Tfh precursors.

In addition to the percentage of CD45RA^−^CD25^low+^ CXCR5^+^ subpopulation among CD4^+^ T-cells, the percentage of CXCR5^+^ cells among CD25highCD127^low/−^ Treg was also significantly increased in peripheral blood from our patients with CLL compared to HC. Therefore, using newer bioinformatics tools and confirmation by conventional gating, we found significantly higher proportions of CXCR5^+^ cells among Treg in our CLL patients, which could be related to their easier entry into the lymph node TME.

Indeed, the percentage of CXCR5^+^ Treg cells among CD4^+^ T-cells significantly increased in patients with Binet stage B disease, characterized by three or more regions of enlarged lymph nodes/tissues. In addition, the higher proportions of CD45RA^−^CXCR5^+^ Tfr cells among Treg cells were significantly associated with lower TD value, representing predominantly lymph node involvement.

In the referenced study that identified a CD38^+^ subset of germinal center (GC)-resident, T follicular helper (Tfh)-derived regulatory T-cells (Tfr) in human tonsils, the authors demonstrated that this subset not only retained the ability to support B cell responses but also exhibited suppressive functions [50]. A more recent investigation extended these findings to the peripheral blood, showing that circulating Tfr cells can be classified based on CD38 expression. Notably, CD38^+^ circulating Tfr cells displayed phenotypic similarities to their tonsillar counterparts [51]. In this latter study, PD-1 blockade led to an expansion of the CD38^+^ circulating Tfr cell population, which was associated with elevated IgG and IgA titers in treated patients. However, no significant correlation was observed between the proportion of CD38^+^ circulating Tfr cells and serum immunoglobulin levels in healthy controls or untreated individuals.

However, a significantly lower percentage of CD38^+^ cells among CD45RA^−^ CXCR5^+^ Tfr found in our patients with CLL compared to HC could impact plasmablast differentiation and the associated early antibody response [51,52]. On the other hand, significantly decreased PD-1 expression on Tfr cells from our patients could make them more suitable for performing their suppressive function [53].

In line with the results of a recent study [54], we found significantly higher PD-1 expression on CD45RA^−^CXCR5^+^ Tfh from our patients with CLL compared to HC. Of note, previous studies on Tfh in patients with CLL either did not analyze Tfr [12,54,55] or only reported a percentage of Tfr as CD25^+^ CD127^−^ cells among Tfh [56].

In our patients, who did not receive any therapy at the time of analysis, phosphorylation of STAT3, a potent mediator of IL-10-driven anti-inflammatory signaling [57], was not significantly different compared to HC.

Of note is that the IL-10 transcript is upregulated within the CLL cells of the lymph node [9,58], and STAT3 is also phosphorylated in the tissues of CLL patients [9,59,60].

Previous studies have not only described basal/constitutive activation of the JAK2/STAT3 pathway in CLL B lymphocytes, but its inhibition also led to CLL cell death, independent of the protective microenvironment of bone marrow mesenchymal stromal cells [31].

The STAT3 pathway plays a key role in the transduction of signals that enable the transcription of anti-apoptotic genes such as BCL-2 and myeloid cell leukaemia one antigen (MCL-1), which are also overexpressed in CLL. Another cytokine, IL-6 in CLL, acts via activation of JAK2 in conjunction with signaling from BCR [31]. T lymphocytes with high BCL-2 expression have recently been described in CLL patients, and this was especially true for Treg [61]. Therefore, enhanced STAT3 signaling could contribute to high Bcl-2 expression in treated patients, as we observed significantly higher levels of basal STAT3 phosphorylation in CD4^+^ T-cells from patients on therapy compared to untreated patients with CLL. Basal pSTAT3 levels were not significantly higher in patients treated with CIT than in patients on BTKi therapy, suggesting that cytokine STAT3 signaling may not depend on the type of therapy.

The role of IL-7 dependent STAT5 signaling in nTreg homeostasis was shown before [62]. The nTreg pSTAT5 signaling response, associated with an increase in aTreg/nTreg ratio, which was more significant in samples from our CLL patients during SARS-CoV-2 antigen-specific stimulation, suggests the role of IL-2-dependent STAT5 signaling in the differentiation of nTreg into aTreg.

Patients with CLL are particularly vulnerable to infections, which remain the leading cause of death in this population [63]. They also face an increased risk of experiencing more severe forms of COVID-19 and have higher associated mortality rates [64,65]. In our previous study [29], we identified a subgroup of patients with elevated proportions of activated regulatory T-cells (aTregs) among CD4^+^FOXP3^+^ T-cells at the initiation of therapy; this group experienced more frequent episodes of severe infections during follow-up. Although in the present study, no significant associations with disease course/severe infections and the frequency of the nTreg subset were found, a decrease in the percentage of nTreg among patients’ CD4^+^FOXP3^+^ T-cells correlated with a longer disease duration. nTreg cells differentiate into aTreg cells upon antigen/T-cell receptor stimulation [30]. As we found no significant correlation between aTreg and disease duration, nTreg depletion with prolonged disease process may not be associated with their differentiation into aTreg.

Among transcription factors that have a crucial influence in determining Treg fate at the transcription level, BTB Domain and CNC Homolog 2 (BACH2) are downregulated in aTreg cells but are highly expressed in resting/naïve Treg cells and promote their quiescence and long-term maintenance [66,67]. Increased aTreg proportions in advanced disease and decreased rTreg with a longer disease duration suggest the possible role of decreased BACH2 expression, which was described recently in patients with CLL [68,69]. As the incidence and severity of hypogammaglobulinemia also increase with disease duration [70], the relationship between nTreg and Tfr, implicated in suppression of humoral immune responses in secondary lymphoid follicles [47], warrants further study. Of note, BACH2 was also shown to attenuate IL-2 signaling and promote the development of highly differentiated Tfr cells [71]. Finally, CD38, which is an important therapeutic target in multiple myeloma [72] and possibly also in CLL [73], was among the Treg cell signature genes dysregulated in Bach2-deficient T-cells [74], suggesting that decreased expression of BACH2 in CLL could also be related to the decrease in the CD38^+^ subset of Tfr found in our patients. In addition, the basic leucine zipper transcription factor ATF-like (BATF) is induced by TCR stimulation during aTreg differentiation [74]. Of note, FoxP3 expression and Treg stability require continuous BATF expression in Tregs, as it regulates demethylation and accessibility of the CNS2 region of the *Foxp3* locus [66].

## Figures and Tables

**Table 1:** Demographic, clinical, and laboratory data at the time of study entry.

Parameter	Group1 ^a^	Group2 ^a^	*p*	Adjusted *p*
Cohort size	20	19	NA	NA
Age (years)	69 (3)	67 (2)	0.46	NS
Gender	7 F/13 M	6 F/13 M	>0.99	NS
Ethnicity	20 Slovene	19 Slovene	NA	NA
Binet stage C	12/20	15/19	0.35	NA
Disease duration (months)	39 (3)	69 (3)	0.0153	NS
Age at diagnosis (y)	66 (3)	62 (2)	0.12	NS
TTM score t0	15.9 (1.4)	17.1 (2.1)	0.84	NS
TD score t0	0.76 (0.04)	0.76 (0.05)	0.99	NS
Lymphocytes t0 (×10^9^/L)	143.5 (20.4)	164.2 (27.8)	0.82	NS
Neutrophils t0 (×10^9^/L)	3.3 (0.5)	3.9 (0.5)	0.36	NS
CD4 count t0 (×10^3^/L)	2309 (306)	1971 (229)	0.53	NS
CD4% t0 (%)	3.3 (1.2)	2.2 (0.6)	0.44	NS
AIHA	1/20	3/19	0.34	NS
Preexisting CLL therapy	4/20	8/19	0.18	NS
Hgb t0 (g/L)	110 (6)	101 (5)	0.28	NS
Tr t0 (×10^9^/L)	141 (19)	135 (13)	0.62	NS

^a^ *p* value refers to the comparison of group 1 (nTreg ≥ 9.6% among CD4^+^FOXP3^+^ T-cells) vs. group 2 (nTreg < 9.6% CD4^+^FOXP3^+^ T-cells); adjusted *p*, Bonferroni-adjusted *p* value; CD4%, percentage of CD4^+^ T-cells among lymphocytes; AIHA, autoimmune hemolytic anaemia; F, female; M, male; NA, not applicable; NS, *p* > 0.05; pre-existing CLL therapy, any therapy of CLL before enrollment; Hgb t_0_, hemoglobin concentration at time of enrollment; t_0_, at time zero enrolment; ^a^ mean (SEM) or the number of patients/entire group.

**Table 2:** Therapy during follow-up.

Therapy	Combinations	Group1 *^a^*		Group2 *^a^*		*p*	Adjusted *p*
		*n*/*N*	%	*n*/*N*	%		
CIT	All	6/20	30	6/19	32	>0.99	NS
	FCR	2/20	10	2/19	10	>0.99	NS
	Chlorambucil + Rituximab	4/20	20	2/19	10	0.66	NS
	Chlorambucil + Obinutuzumab	0/20	0	1/19	5	>0.99	NS
	Bendamustine + Rituximab	0/20	0	1/19	5	>0.99	NS
BTKi	All	12/20	60	9/19	47	>0.99	NS
	Ibrutinib	5/20	25	6/19	32	0.73	NS
	Acalabrutinib	6/20	30	2/19	10	0.23	NS
	Acalabrutinib + Obinutuzumab	1/20	5	1/19	5	>0.99	NS
Venetoclax	All combinations	2/20	10	4/19	21	0.41	NS
	+Rituximab	0/20	0	2/19	10	0.23	NS
	+Obinutuzumab	0/20	0	1/19	5	>0.99	NS
	+Bendamustine + Obinutuzumab	2/20	10	1/19	5	>0.99	NS

*^a^* No. of patients/whole group; *p* value refers to the comparison of group 1 (nTreg ≥ 9.5% of CD4^+^FOXP3^+^ T-cells) vs. group 2 (aTreg < 9.5% of CD4^+^FOXP3^+^ T-cells); adjusted *p*, Bonferroni adjusted *p* value; therapy given at start of and during follow-up; FCR, Fludarabine+Ciclophosphamide+Rituximab combination; NS, *p* > 0.05.

## Data Availability

Datasets used in this article are available from the corresponding author upon reasonable request.

## Supplementary Materials

The following supporting information can be downloaded at: https://www.mdpi.com/article/10.3390/biomedicines13051204/s1, Figure S1: (A) gating for identification of CD45RA^+^ FOXP3lo nTreg, CD45RA^−^ FOXP3hi aTreg, and CD45RA^−^ FOXP3lo non-Treg subsets cells among FOXP3^+^CD4^+^ T-cells; (B) cumulative percentages of CXCR5^+^ cells among non-Treg, aTreg and nTreg cells from healthy controls (white bars), and patients with CLL (gray bars); (C) correlation between CD25^+^ CD127^low/−^ Treg and the sum of percentages of aTreg and nTreg subsets among CD4^+^ T-cells. Figure S2: representative histograms and cumulative data on PD-1 expression in both the Tfr and Tfh subsets of CD4^+^ T-cells from CLL patients and HC. Figure S3: pSTAT3 levels in whole-blood samples were collected and incubated/stimulated in tubes from the Quantiferon SARS-CoV-2 kit for 16–24 h, as described in the Materials and Methods. Representative histograms of pSTAT3 in gated CD25^+^FOXP3^+^CD4^+^ T-cells from the same healthy donor whole-blood aliquots withdrawn from negative control (Nil) and from the two Ag tubes mixed together (+Ag), incubated with anti-IL2 neutralizing antibodies or left untreated, are shown. Figure S4: (A) Survival curve (Kaplan–Meier plot) showing time to first grade ≥ 3 infection in group 2 patients with CLL with lower nTreg percentages (<9.6%) among CD4^+^FOXP3^+^ T-cells at start of follow-up compared to group 1 patients with CLL with higher nTreg percentages (≥9.6%) (B) Disease courses of 39 patients with CLL (y axis) followed up to 500 days. The color of dotted lines reflects subgroup designations (group 1 black and group 2 red dashed lines). Supplementary Table S1. The characteristics of recruited patients with CLL are not included in the follow-up. Supplementary Table S2. The characteristics of recruited healthy subjects for the determination of CD38^+^ cells among Treg subsets, Ab. Titers and Treg subsets.
