# Supplementary material for: Increased STAT3 Phosphorylation in CD4+ T-Cells of Treated Patients with Chronic Lymphocytic Leukemia and Changes in Circulating Regulatory T-Cell Subsets Relative to Tumor Mass Distribution Value and Disease Duration
Source: Biomedicines. 2025 May 15;13(5):1204. doi: 10.3390/biomedicines13051204 (PMC12109142; doi:10.3390/biomedicines13051204)
Supplement: Supplementary file 1 [file biomedicines-13-01204-s001.zip › Suppl. table S1.pdf]

Supplementary Table S1: Characteristics of CLL patients not included in the follow up

	Gender	Age	Disease stage	Previous therapy	AIHA
Patient 1	male	62	Binet A	0	No
Patient 2	male	74	Binet A	0	No
Patient 3	male	61	Binet A	0	No
Patient 4	male	64	Binet A	0	No
Patient 5	male	70	Binet A	0	No
Patient 6	male	73	Binet B	0	No
Patient 7	female	69	Binet B	0	No
Patient 8	male	69	Binet A	0	Yes
Patient 9	male	72	Binet A	0	No
Patient 10	male	83	Binet A	0	No
Patient 11	male	80	Binet A	0	No
Patient 12	male	80	Binet A	0	No
Patient 13	male	69	Binet C	0	No
Patient 14*	female	78	Binet A	BTKi	No
Patient 15	male	71	Binet A	0	No
Patient 16*	male	56	Binet A	CIT	No
Patient 17*	male	81	Binet A	BTKi	No
Patient 18	male	84	Binet A	0	No
Patient 19	female	69	Binet A	0	No
Patient 20	female	66	Binet A	0	No
Patient 21	male	78	Binet A	CIT	Yes
Patient 22	male	83	Binet B	0	No
Patient 23	male	71	Binet B	0	No

Abbreviations: AIHA; Autoimmune Hemolytic Anemia; CIT, chemo-immunotherapy; BTKi, Bruton Tyrosine Kinase inhibitor, * patients treated before enrollment
