# Supplementary material for: Increased STAT3 Phosphorylation in CD4+ T-Cells of Treated Patients with Chronic Lymphocytic Leukemia and Changes in Circulating Regulatory T-Cell Subsets Relative to Tumor Mass Distribution Value and Disease Duration
Source: Biomedicines. 2025 May 15;13(5):1204. doi: 10.3390/biomedicines13051204 (PMC12109142; doi:10.3390/biomedicines13051204)
Supplement: Supplementary file 1 [file biomedicines-13-01204-s001.zip › Suppl. table S2.pdf]

Supplementary Table S2: Characteristics of healthy controls included in the study

	Gender	Age	Al., A.I., A.D.*	Immunosuppressive therapy
Control 1	female	49	No	0
Control 2	male	39	No	0
Control 3	female	52	No	0
Control 4	female	53	No	0
Control 5	female	41	No	0
Control 6	male	50	No	0
Control 7	female	55	No	0
Control 8	female	47	No	0
Control 9	male	29	No	0
Control 10	male	73	No	0
Control 11	female	70	No	0
Control 12	female	44	No	0
Control 13	female	46	No	0
Control 14	female	59	No	0
Control 15	female	47	No	0
Control 16	female	59	No	0
Control 17	female	48	No	0
Control 18	female	65	No	0
Control 19	female	44	No	0
Control 20	female	48	No	0
Control 21	female	38	No	0
Control 22	female	59	No	0
Control 23	female	48	No	0
Control 24	female	60	No	0
Control 25	female	42	No	0
Control 26	female	31	No	0
Control 27	female	67	No	0
Control 28	female	26	No	0
Control 29	female	50	No	0
Control 30	female	38	No	0
Control 31	female	55	No	0
Control 32	female	47	No	0

Healthy controls included in the study for determination CD38+ subset of aTreg and Tfr 14 days after second dose of the BNT162b2 mRNA COVID-19 vaccination, along with determination of IgG antibodies against SARS-CoV-2 Spike protein 3 months after second dose of the BNT162b2 mRNA COVID-19 vaccination.

Abbreviations: Al., Allergy; A.I., Acute infection; A.D., Autoimmune Disease; *

	Gender	Age	Al., A.I., A.D.*	Immunosuppressive therapy
Control 1	female	54	No	0
Control 2	male	76	No	0
Control 3	female	58	No	0
Control 4	female	73	No	0
Control 5	female	56	No	0
Control 6	female	55	No	0
Control 7	female	54	No	0
Control 8	female	83	No	0
Control 9	female	54	No	0
Control 10	female	58	No	0
Control 11	female	61	No	0
Control 12	female	57	No	0
Control 13	female	54	No	0
Control 14	female	63	No	0
Control 15	male	52	No	0
Control 16	male	53	No	0
Control 17	female	63	No	0
Control 18	female	51	No	0
Control 19	female	54	No	0
Control 20	male	78	No	0

Healthy controls included in the study for determination of Treg subsets.

Abbreviations: Al., Allergy; A.I., Acute infection; A.D., Autoimmune Disease; *
